# Statistical study of bacteremia secondary to catheter (BSC) in our intensive care unit (ICU). bacteremia zero (BZ) program implementation

**DOI:** 10.1186/2197-425X-3-S1-A883

**Published:** 2015-10-01

**Authors:** R Fernández Fernández, MR Ramírez Puerta, O Moreno Romero, ME Yuste Ossorio, M Muñoz Garach

**Affiliations:** Unidad Cuidados Intensivos, Hospital Universitario San Cecilio, Granada, Spain

## Introduction

The BSC infections increase mortality, complications, hospital stay and the costs. In our setting, BSC is one of the device-associated infections more common. The Alliance for Patient Safety, WHO, launched in 2004, the campaign for the prevention of infection associated to medical care though hand hygiene. The Working Group on Infectious Diseases of the Spanish Society of Intensive Care Medicine and Coronary Units (GTEI-SEMICYUC), developed the National Survey of Nosocomial Infection Surveillance (ENVIN) as a computerized record of the incidence of nosocomial infection for services or intensive care units (ICU). Were selected for monitoring those most serious and frequent nosocomial infections related to instrumentation, including ventilator-associated pneumonia , urinary tract infection associated with urethral catheterization and BSC

## Objectives

We want to show the evolution of BSC: central venous catheter (CVC) and arterial (CA) in our 18-bed unit, after the implementation of BZ program and compare it with spanish data.

## Methods

- Data from ENVIN 01.04.11 / 01.04.2015. 18 ICU beds.

Rates: BSC/100 admitted, BSC/CVC-CA. Incidence density (ID): BSC/1000ds,

BSC/1000 days CVC-CA

N 4198 (patients admitted to ICU), 1659 patients with CVC-CA, 17575 days of stay

(ds),11024 days of CVC, CA.

- Training and educational campaign were imparted to professionals of our unit and

Anesthesiology service (235 people). Some classes were presencial and another were by means of internet. Implementation of protocol for hand washing, technical skills about the insertion of CVC, cleaning and maintenance of catheters.

- The main objective of BZ project was to reduce BRC DI < 4 episodes per 1000 CVC

days

## Results

01.04.11-01.04.12: 3 BSC 0.29/100 admitted, 0.80/CVC-CA, 0.62/1000ds, 0.96/1000

days CVC-CA. Germ A. Baumanii 33.33%, P.mirabilis 33.33%, S. Epidermidis

33.33%. Sepsis 66.67%

01.04.12-01.04.13: 7 BSC. 0.65/100 admitted, 1.61/CVC-CA, 1.42/1000ds, 2.1/1000

days CVC-CA. Germ S. Coagulasa negativo (SCN). Severe sepsis 28.57%

01.04.13-01.04.14: 5 BSC 0.44/100 admitted, 1.08/CVC-CA, 1.02/1000ds, 1.63/1000

days CVC-CA. Germ S. Epidermidis. Sepsis 60%

01.04.14-01.04.15: 3 BSC 0.31/100 admitted, 0.78/100 CVC-CA, 0.71/1000 ds,

1.07/1000 days CVC-CA. Germ: S. epidermidis. Sepsis 100%.

Spain, last year: N = 20799, 1.59/100 admitted, 2.51/100 pat CVC-CA, 2.10/1000 ds,

1.69/ 1000 days CVC-CA. Germ S. Epidermidis. Sepsis 64.85%

## Conclusions

We are below the level of BSC set by the SEMICYUC with fewest BSC than the rest of the country through the implementation of the program BZ. In the last year, we have improved our numbers because the realization of program recommendations has been monitored more intensively since in the previous two years had been a relaxation and thereby increased the numbers of BSC.

## Grant Acknowledgment

UCI San Cecilio
